# MITIGATION OF MAJOR HIP INJURY FROM FALLS: EARLY RESEARCH RESULTS WITH SMART BELT STUDY

**DOI:** 10.1093/geroni/igad104.3477

**Published:** 2023-12-21

**Authors:** Rebecca Tarbert

**Affiliations:** Active Protective Technologies, Inc, Fort Washington, Pennsylvania, United States

## Abstract

Hip fractures sustained from falling are a devastating outcome for over 300,000 older adults every year in the US. The recovery from this major injury is extremally costly to the healthcare system and results in reduced mobility, increased dependency and up to 30% higher morbidity within 12 months of fracture. Avoidance of falls and injuries from falls is embedded into the standard of care for older adult providers though these standardized measures have not reduced the rate of death from falls as shown in recent studies. The study, Mitigation of Major Hip Injury due to fall in an At-Risk, Older Adult Population with a Wearable Smart Belt, seeks to compare the safety and efficacy of a wearable smartbelt to be worn around the waist of high risk older adults in order to mitigate major hip injuries related to falls through built-in sensors and the deployment of anatomically situated airbags around the hips during the hip impacting fall. The design of the study includes multiple older adult settings where subjects who are identified as being at high risk of fragility fracture and falls wear the study device for 6 months. Comparison of falls with major hip injury, emergency room visits and hospitalizations from falls and hip fractures from falls will be compared to a retrospective control group matched with the same inclusion/exclusion criteria. The poster will share the subject criteria and data gathered into November 2023. This study is listed on clinicaltrials.gov NCT#05245097

